# α‐Synuclein Promotes Atherosclerosis by Impairing Macrophage Autophagic Flux

**DOI:** 10.1111/acel.70713

**Published:** 2026-09-28

**Authors:** Mengting Qin, Xuying Xiang, Xiaoqing Guo, Shengnan Wang, Jiaojiao Chen, Dailiang Jiang, Yuhang Feng, Jiangnan Yu, Ling Mao

**Affiliations:** ^1^ Department of Neurology, Union Hospital, Tongji Medical College Huazhong University of Science and Technology Wuhan China; ^2^ Department of Rehabilitation, Union Hospital, Tongji Medical College Huazhong University of Science and Technology Wuhan China

**Keywords:** atherosclerosis, autophagy, FLOT1, α‐Synuclein

## Abstract

Atherosclerosis, recognized as a prototypical age‐associated vascular disease, is driven by macrophage foam cell formation. Individuals with Parkinson's disease (PD) exhibit an elevated risk for stroke. While α‐synuclein (α‐Syn) is known for its neuronal role in PD, its presence in the circulation and potential vascular effects remain unexplored. Here, we identify α‐Syn as a previously unrecognized circulating factor that promotes atherosclerosis. α‐Syn was detected in human atherosclerotic plaques. Genetic deletion of α‐Syn reduced atherosclerotic lesion size and macrophage lipid accumulation in a mouse model of atherosclerosis induced by overexpression of proprotein convertase subtilisin/kexin type 9 (PCSK9). On the contrary, administration of exogenous α‐Syn accelerated atherosclerosis in Apolipoprotein E knockout (*Apoe* ^−/−^) mice. Mechanistically, α‐Syn promoted macrophage foam cell formation by impairing autophagic flux, accompanied by activation of PI3K‐mTOR signaling. Proteomic analysis identified the membrane raft protein flotillin‐1 (FLOT1) as a candidate upstream mediator. *Flot1* knockdown reduced α‐Syn internalization, PI3K phosphorylation, autophagy impairment, and lipid accumulation, while pharmacological inhibition of PI3K or mTOR attenuated the effects of α‐Syn. Together, these findings identify a FLOT1‐associated pathway through which extracellular α‐Syn disrupts macrophage autophagy and promotes atherosclerosis.

## Graphical abstract

Extracellular α‐synuclein activates the FLOT1‐PI3K‐mTOR axis to block autophagosome‐lysosomal fusion and lipophagy, thereby promoting macrophage foaming and atherosclerosis. Knockdown of FLOT1 or inhibition of PI3K‐mTOR rescues these effects. The graphical abstract image was created with Figdraw.

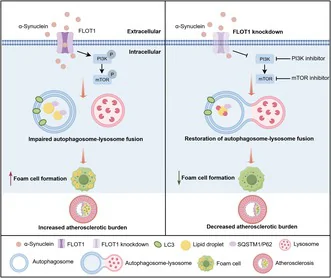

## 1 Introduction

Atherosclerosis is a prototypical age‐associated vascular disease (Wang and Bennett 2012) and the pathological basis of most cardiovascular events (Libby et al. 2011). A key early event in this process is the formation of macrophage‐derived foam cells, which arises from an imbalance between lipid uptake, intracellular degradation, and cholesterol efflux (Gui et al. 2022; Moore and Tabas 2011). Beyond classical lipid‐handling pathways, the autophagy‐lysosome system has emerged as a critical regulator of intracellular lipid homeostasis in macrophages. Through lipophagy, lipid droplets are delivered to lysosomes for hydrolysis, thereby generating free cholesterol for cellular efflux (Laval and Ouimet 2023; Shao et al. 2016). Impairment of autophagic flux promotes lipid droplet accumulation and foam cell formation, and age‐related decline in autophagy function is increasingly recognized as an important contributor to vascular pathology (Aman et al. 2021; Boucher et al. 2025). However, the upstream factors that disrupt macrophage autophagy in atherosclerosis remain incompletely understood.

α‐Synuclein (α‐Syn), a small cytosolic protein best known for its role in Parkinson's disease (PD) and related synucleinopathies (Burre et al. 2018), has recently attracted attention beyond the central nervous system (CNS). Although highly enriched in neurons, α‐Syn is also detectable in extracellular fluids and peripheral tissues (Burre et al. 2018; Magalhaes and Lashuel 2022), and circulating α‐Syn levels are elevated in patients with PD (Zubelzu et al. 2022). These findings support the potential utility of blood α‐Syn as a peripheral biomarker for PD, while also raising the hypothesis of whether elevated circulating α‐Syn exerts additional biological effects outside the CNS. Notably, epidemiological studies have suggested that PD is associated with an increased burden of atherosclerotic vascular disease, including a higher prevalence of carotid plaques (Alves et al. 2021) and an increased risk of ischemic stroke (Park et al. 2020), pointing to a potential link between α‐Syn‐related pathology and vascular aging.

Several lines of evidence further support the plausibility that α‐Syn may influence lipid metabolism. α‐Syn has been detected in high‐density lipoprotein (HDL) and lipoprotein(a) fractions, indicating potential interactions with circulating lipoproteins (Emamzadeh and Allsop 2017). Its amphipathic α‐helical domain and cholesterol‐binding capacity enable association with lipid membranes, and previous studies have implicated α‐Syn in vesicular trafficking (Fantini et al. 2011), intracellular cholesterol metabolism (Sarchione et al. 2021), and lipid droplet (LD) accumulation (Cole et al. 2002; Outeiro and Lindquist 2003). These properties suggest that α‐Syn may participate in lipid‐handling processes relevant to foam cell formation. However, whether α‐Syn directly regulates macrophage lipid metabolism and thereby contributes to atherogenesis remains unknown.

Disruption of autophagy is a well‐recognized feature of α‐Syn‐associated cellular pathology. Accumulating evidence indicates that α‐Syn can interfere with autophagy at multiple levels (Bayati and McPherson 2024; Pei et al. 2024), although the specific mechanisms appear to be highly context‐ and cell type‐dependent. Importantly, despite extensive studies in neuronal systems (Tu et al. 2021). Most previous work has focused on neuronal systems, leaving it unclear whether α‐Syn alters autophagic flux in macrophages.

In this study, we assessed the effects of α‐Syn on atherosclerotic lesion development in mouse models and on macrophage lipid accumulation in vitro. Mechanistically, we examined whether α‐Syn impairs autophagic flux by disrupting autophagosome‐lysosome fusion and evaluated the upstream regulator linking extracellular α‐Syn to intracellular signaling and autophagy dysfunction. Together, our findings identify α‐Syn promotes macrophage foam cell formation and atherosclerosis and suggest a mechanistic connection between synuclein‐associated pathology and vascular aging.

## 2 Results

### 2.1 SNCA Knockout Mice Exhibit Attenuated Atherosclerosis

To investigate the relationship between α‐Syn and atherosclerosis, we asked whether α‐Syn is present in human atherosclerotic lesions. Atherosclerotic plaque specimens were collected from three patients undergoing internal carotid endarterectomy and subjected to liquid chromatography–tandem mass spectrometry (LC‐MS/MS) analysis to detect protein peptide segments (Figure 1a). Peptides corresponding to α‐Syn were detected in all three specimens (Figure S1a). Consistent with the proteomic findings, western blotting (WB) revealed an α‐Syn‐immunoreactive band migrating slightly above 15 kDa, at a position comparable to that of the recombinant human His‐tagged α‐Syn monomer (Figure S1b). Additional α‐Syn‐immunoreactive bands were observed at approximately 25 and 55 kDa (Figure S1b). These higher‐molecular‐weight bands may represent post‐translationally modified or protein‐associated forms of α‐Syn. Collectively, these results support the presence of α‐Syn in human atherosclerotic plaques and provide protein‐level validation of the LC‐MS/MS findings.

**FIGURE 1:**
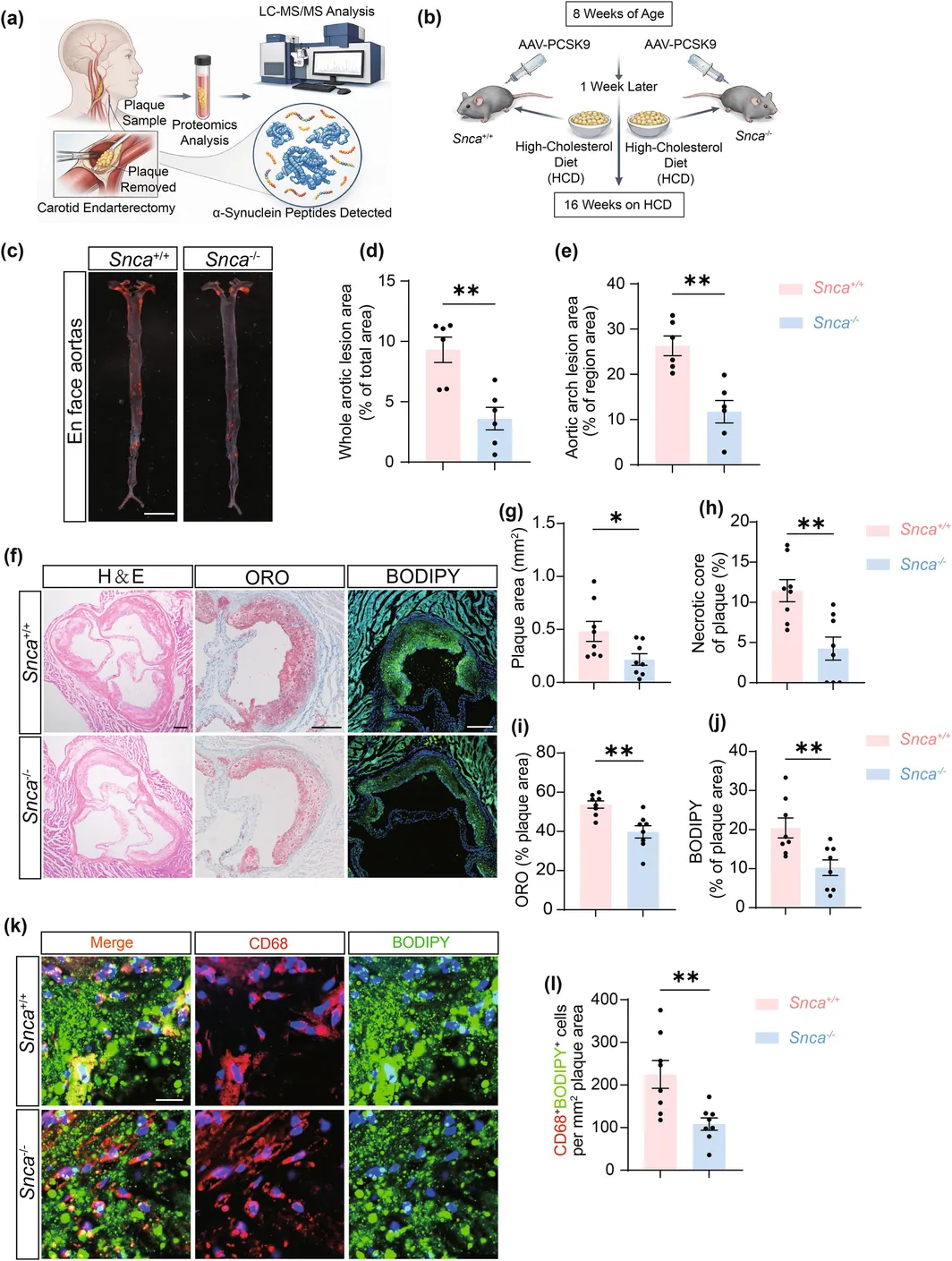
*Snca* deficiency attenuates atherosclerotic lesion development in the AAV‐PCSK9 model. (a) Workflow for human carotid plaque collection and proteomic profiling; α‐Syn‐derived peptides were identified. (b) Schematic of the atherosclerosis induction protocol in male *Snca* ^−/−^ mice and *Snca* ^+/+^ littermate controls. Mice received a single tail‐vein injection of AAV‐PCSK9 followed by 16 weeks of HCD feeding. (c–e) Representative en face ORO staining of the aorta (c) with quantification of lesion area in the whole aorta (d) and aortic arch (e) of male *Snca* ^−/−^ and *Snca* ^+/+^ mice (*n* = 6). Scale bar: 5 mm. (f–j) Representative aortic root sections showing plaque morphology by H&E staining and neutral lipid accumulation by ORO and BODIPY staining (f), with quantification of plaque area (g), necrotic core fraction (h), and lipid deposition normalized to plaque area (i, j) (*n* = 8). Scale bar: 200 μm. (k, l) Representative IF images of aortic root plaques stained for neutral lipids (BODIPY) and the macrophage marker (CD68) (k), with quantification of macrophage‐derived foam cells (CD68^+^ BODIPY^+^) density per mm^2^ plaque area (l) (*n* = 8). Scale bar: 20 μm. All data are shown as the mean ± SEM. Statistical analysis was performed using Mann–Whitney *U* test (d), unpaired *t*‐test (e, g, h, i, j) and Welch's *t*‐test (l). *p < 0.05; **p < 0.01.

To determine whether α‐Syn contributes to atherogenesis, we generated *Snca*‐deficient (*Snca* ^−/−^) mice and used wild‐type littermates (*Snca* ^+/+^) as controls. Representative genotyping results are shown in Figure S2. Atherosclerosis was induced by tail‐vein delivery of adeno‐associated virus encoding proprotein convertase subtilisin/kexin type 9 (AAV‐PCSK9) followed by 16 weeks of high‐cholesterol diet (HCD) feeding (Figure 1b). En face oil Red O (ORO) staining revealed significantly smaller lesion areas in the whole aorta and aortic arch of male *Snca* ^−/−^ mice compared with those of *Snca* ^+/+^ (Figure 1c–e). To assess plaque morphology and composition, we analyzed lesions at the aortic root. Hematoxylin–eosin (H&E) staining showed that male *Snca* ^−/−^ mice developed smaller plaques (Figure 1f,g) and exhibited a reduced proportion of necrotic core relative to total plaque area compared with *Snca* ^+/+^ controls (Figure 1f,h). Consistent with the reduced en face lesion burden, neutral lipid staining with ORO or BODIPY at the aortic root demonstrated decreased lipid deposition normalized to plaque area in male *Snca* ^−/−^ mice (Figure 1f,i,j).

Given the known sex dimorphism in atherosclerosis, we repeated these experiments in female mice. Overall, the phenotype was similar to that observed in males, with attenuated atherosclerotic lesion development in females *Snca* ^−/−^ compared with *Snca* ^+/+^ controls in whole aortas (Figure S3a,b) and aorta arch (Figure S3a,c). H&E and ORO staining showed that, compared with female *Snca* ^+/+^ mice, *Snca* ^−/−^ mice exhibited a smaller plaque area (Figure S3d,e), a lower necrotic core fraction (Figure S3d,f), and reduced lipid deposition at the aortic root (Figure S3d,g).

Because plaque lipid accumulation largely reflects foam cell burden, which is primarily derived from macrophages and vascular smooth muscle cells (VSMCs), we next examined the cellular association of neutral lipids within aortic root plaques. Immunofluorescence (IF) staining was performed to visualize neutral lipids with BODIPY and to identify macrophages and VSMCs with CD68 and ACTA2, respectively. The density of CD68^+^ BODIPY^+^ cells within the plaque area was reduced in *Snca* ^−/−^ mice (Figure 1k,l), whereas the density of ACTA2^+^ BODIPY^+^ cells was not detectably different between genotypes (Figure S4a,b). These data suggest that the reduced lipid content in *Snca* ^−/−^ lesions is primarily attributable to decreased macrophage‐derived foam cell accumulation rather than altered VSMC‐associated lipid storage.

Together, these findings indicate that *Snca* deficiency attenuates atherosclerosis, with a prominent reduction in macrophage‐associated lipid accumulation.

### 2.2 α‐Synuclein Promotes Atherosclerosis by Inducing Macrophage Foam Cell Formation

Could the attenuated atherosclerosis which was observed in *Snca* ^−/−^ mice be explained by an intrinsic impairment in macrophage foam cell formation? To address this, we isolated bone marrow‐derived macrophages (BMDMs) from *Snca* ^−/−^ and *Snca* ^+/+^ mice. After differentiation, cells were incubated with or without ox‐LDL for 24 h, followed by ORO staining to quantify intracellular neutral lipid accumulation. Under these conditions, *Snca* ^−/−^ BMDMs displayed an ORO‐positive area comparable to that of *Snca* ^+/+^ BMDMs (Figure 2a,b), suggesting that *Snca* deficiency does not substantially alter the cell‐autonomous foaming capacity of primary macrophages in vitro.

**FIGURE 2:**
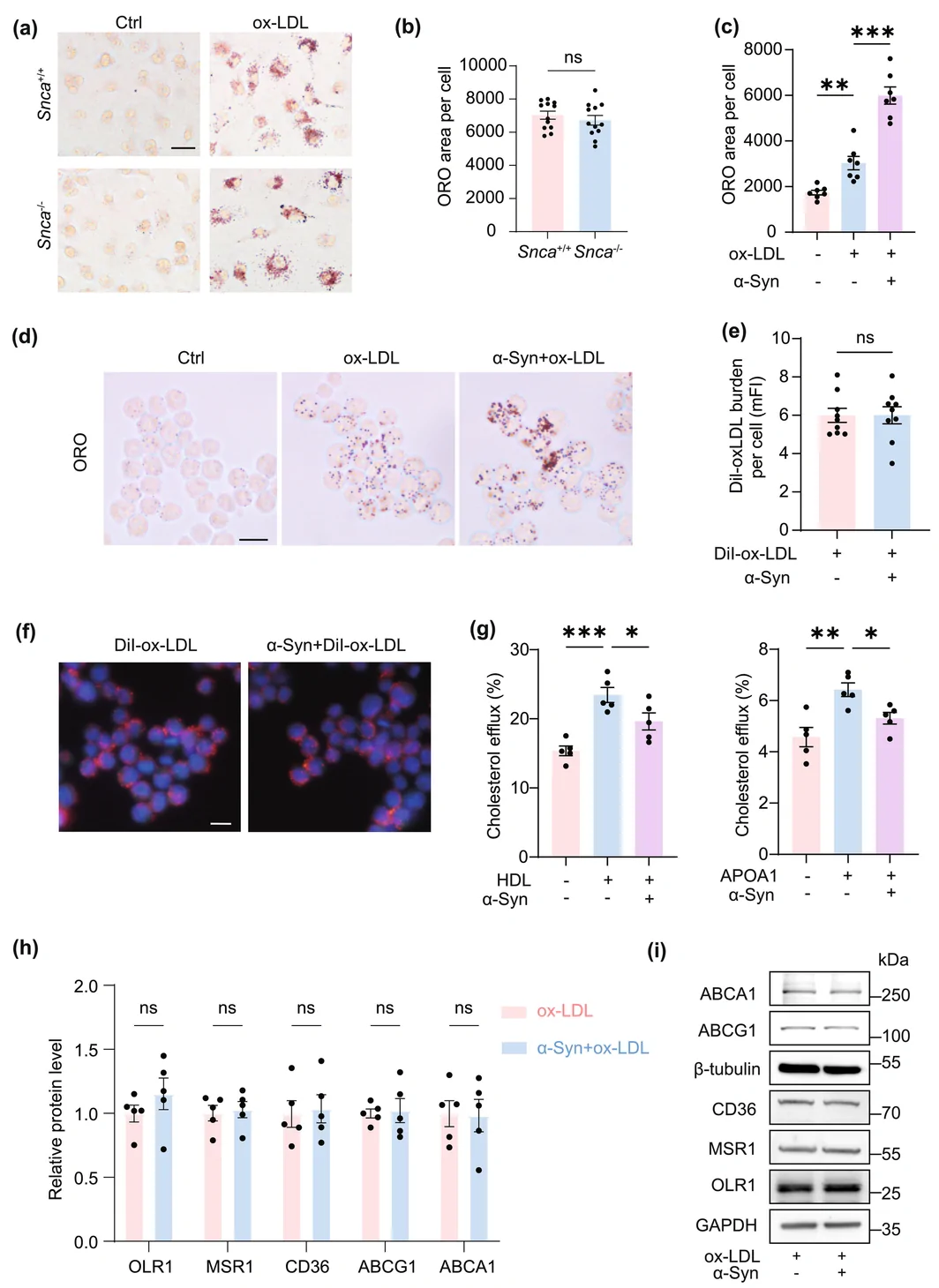
Exogenous α‐Syn promotes macrophage foam cell formation by impairing cholesterol efflux. (a, b) ORO staining images (a) and quantification of ORO‐positive area (b) in BMDMs isolated from *Snca* ^+/+^ and *Snca* ^−/−^ mice after 24 h incubation with or without ox‐LDL (*n* = 12). Scale bar: 20 μm. (c, d) Quantification (c) and representative ORO staining (d) of RAW264.7 treated with vehicle (control), ox‐LDL alone, or α‐Syn plus ox‐LDL for 24 h (*n* = 7). Scale bar: 20 μm. (e, f) IF analysis of DiI‐ox‐LDL uptake in RAW264.7 cells treated with or without α‐Syn (e, n=9) with corresponding representative staning (f). Scale bar: 10 μm. (g) Cholesterol efflux assay using NBD‐cholesterol loading followed by HDL or APOA1‐mediated efflux in the presence or absence of α‐Syn (*n* = 5). (h, i) Densitometric quantification (h) and immunoblotting (i) of classical lipid‐handling receptors and transporters (CD36, MSR1, OLR1, ABCG1, and ABCA1), showing no significant changes upon α‐Syn treatment (*n* = 5). CD36, MSR1, and OLR1 were normalized to GAPDH, whereas ABCG1 and ABCA1 were normalized to β‐tubulin. All data are shown as the mean ± SEM. Statistical analysis was performed using unpaired *t*‐test (b, e, h) and 2‐way ANOVA followed by Dunnett's post hoc test (c, g). ns, *p* > 0.05; *p < 0.05; **p < 0.01; ***p < 0.001.

α‐Syn, although highly enriched in the nervous system, is not limited to nervous tissues (Burre et al. 2018). It is also present in the blood and mostly exists in red blood cells in the form of monomers (Barbour et al. 2008; Fauvet et al. 2012). There is a possibility that α‐Syn in peripheral blood promotes the formation of macrophage foaming. Recombinant mouse His‐α‐Syn monomer was purified as previously described (Volpicelli‐Daley et al. 2014), and its purity was verified by Coomassie brilliant blue staining (Figure S5). To determine whether α‐Syn promotes macrophage foam cell formation, RAW264.7 macrophages were treated with ox‐LDL alone, α‐Syn plus ox‐LDL, or left untreated as a control for 24 h. ORO staining showed that lipid accumulation was lowest in untreated cells, increased after ox‐LDL treatment, and was further enhanced by α‐Syn co‐treatment (Figure 2c,d), indicating that α‐Syn exacerbates macrophage foam cell formation. Lipid accumulation in macrophages could be caused by changes in lipid uptake or cholesterol efflux. Hence, we first examined DiI‐ox‐LDL uptake. After 4‐h incubation, the intracellular DiI‐oxLDL fluorescence was unaffected by α‐Syn treatment (Figure 2e,f). We next examined cholesterol efflux. In macrophages pre‐loaded with NBD‐labeled cholesterol, α‐Syn significantly inhibited cholesterol efflux to both HDL and apolipoprotein A‐1 (APOA1) acceptors, compared to the control condition (Figure 2g). However, the protein levels of the five classical lipid‐handling receptors and transporters, including CD36, macrophage scavenger receptor 1 (MSR1), oxidized low‐density lipoprotein receptor 1 (OLR1/LOX‐1), ATP binding cassette subfamily G member 1 (ABCG1), and ATP binding cassette subfamily A member 1 (ABCA1), were not affected (Figure 2h,i). This suggests that α‐Syn might affect lipid efflux in another way.

To assess the pro‐atherosclerotic effect of α‐Syn in vivo, we utilized male *Apoe*‐deficient (*Apoe* ^−/−^) mice as a model of atherosclerosis. Age‐ and weight‐matched mice received either α‐Syn or PBS via tail vein injection. After 12 weeks on HCD feeding, the aorta and other tissues were collected. En face ORO staining revealed a marked increase in lipid deposition in both the whole aorta and the aortic arch of α‐Syn‐treated mice compared to PBS‐treated controls (Figure 3a–c). H&E staining of the aortic root revealed that α‐Syn‐treated mice exhibited larger plaque sizes and an increased percentage of the necrotic core area (Figure 3d–f). Consistently, the area of lipid deposition, as visualized by ORO and BODIPY staining in the aortic root, was also significantly expanded in the α‐Syn group (Figure 3h–j). Masson's trichrome staining indicated no significant difference in collagen content between the two groups (Figure 3g,h). These findings demonstrate increased lipid content and decreased plaque stability in the α‐Syn treatment group, indicating more severe atherosclerotic lesions.

**FIGURE 3:**
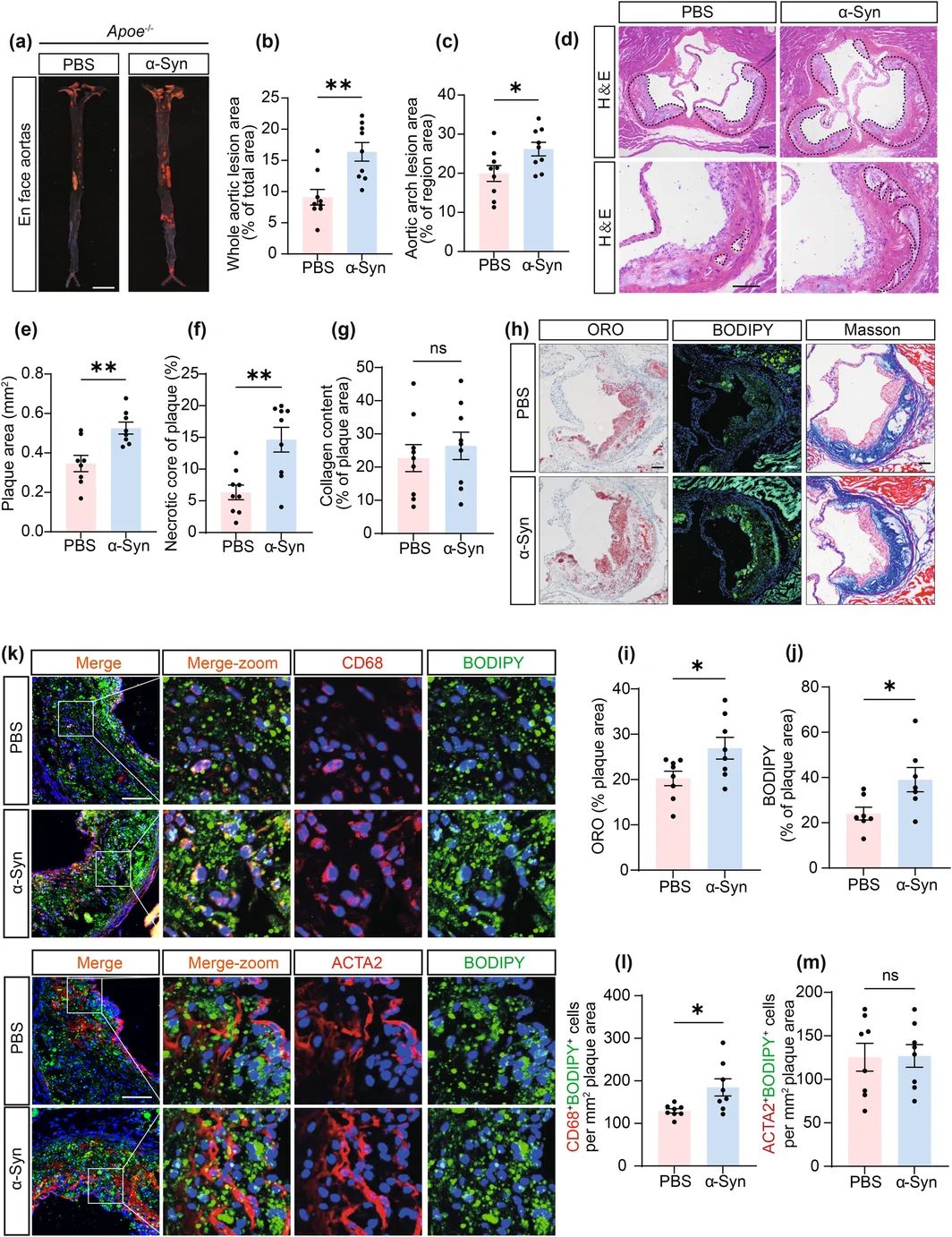
Peripheral administration of α‐Syn increases plaque lipid burden and macrophage foam cell formation in *Apoe* ^−/−^ mice. (a–c) Representative en face ORO staining of whole aortas and aortic arches from *Apoe* ^−/−^ mice receiving repeated tail‐vein injections of α‐Syn or PBS during 12 weeks of HCD feeding (a), with quantification of lesion area in the whole aorta (b) and aortic arch (c) (*n* = 9). Scale bar: 5 mm. (d–f) Representative H&E‐stained aortic root sections (d) with quantification of plaque area (e) and necrotic core fraction (f) (*n* = 8–9). Scale bar: 100 μm. (g, h) Representative Masson's trichrome staining and quantification of collagen content (*n* = 9). Scale bar: 100 μm. (h–j) Assessment of plaque lipid accumulation by ORO and BODIPY staining (h) with quantification of lipid deposition (*n* = 7–8) (i, j). Scale bar: 100 μm. (k–m) Representative IF images of aortic root plaques stained for neutral lipids (BODIPY) and macrophages or VSMCs (k) and quantification of CD68^+^ BODIPY^+^ cells and ACTA2^+^ BODIPY^+^ cells density per mm^2^ plaque area (l, m) (*n* = 8). Scale bar: 100 μm. All data are shown as the mean ± SEM. Statistical analysis was performed using Mann–Whitney *U* test (b), unpaired *t*‐test (c, e–g, i, j, m) and Welch's *t*‐test (l). ns, *p* > 0.05; *p < 0.05; **p < 0.01.

We next investigated the effect of α‐Syn on macrophage foam cell formation in vivo by assessing the co‐localization of BODIPY with cell‐type markers. The α‐Syn‐treated group exhibited a higher density of BODIPY^+^ CD68^+^ cells per unit plaque area (Figure 3k,l), suggesting increased macrophage‐derived foam cell accumulation. In contrast, α‐Syn did not affect the lipid deposition in VSMC‐derived foam cells (Figure 3k,m), suggesting no detectable effect on VSMC‐associated lipid accumulation.

The above results indicate that α‐Syn promotes macrophage foam cell formation and accelerates the progression of atherosclerosis.

### 2.3 α‐Synuclein Disrupts Autophagosome‐Lysosome Fusion in Macrophages

Given that the autophagy‐lysosome system is one of the important pathways for intracellular lipid degradation, impaired autophagic function can lead to insufficient lipid clearance and accumulation of cytoplasmic LDs (Zechner et al. 2017), we therefore quantified the protein expression of microtubule associated protein 1 light chain 3 (MAP1LC3/LC3) and sequestosome 1 (SQSTM1/p62) to confirm the autophagic alterations (Ke 2024; Klionsky 2007). In macrophages treated with ox‐LDL for 24 h, α‐Syn treatment led to the accumulation of LC3B‐II and SQSTM1. Notably, upon pre‐treatment with chloroquine (CQ), a lysosomal inhibitor, LC3B‐II levels no longer increased, while SQSTM1 levels remained increased (Figure 4a,b). The CQ‐based autophagy flux assay suggests that increase of LC3B‐II might be due to the blockage at the degradation stage of autophagy. Consistently, compared with the control group, α‐Syn treatment did not significantly alter *Lc3b* mRNA expression, whereas *Sqstm1* mRNA levels were increased (Figure S6). It indicates that the accumulation of LC3B‐II is not due to transcriptional upregulation, while the accumulation of SQSTM1 may reflect both increased synthesis and reduced degradation. Similarly, compared with the PBS‐treated controls, *Apoe* ^−/−^ mice receiving α‐Syn showed more LC3^+^ CD68^+^ (Figure S7a,b) and SQSTM1^+^ CD68^+^ (Figure S7c,d) cells within aortic root plaques. Taken together, these observations are consistent with a disturbance in autophagic flux following α‐Syn treatment.

**FIGURE 4:**
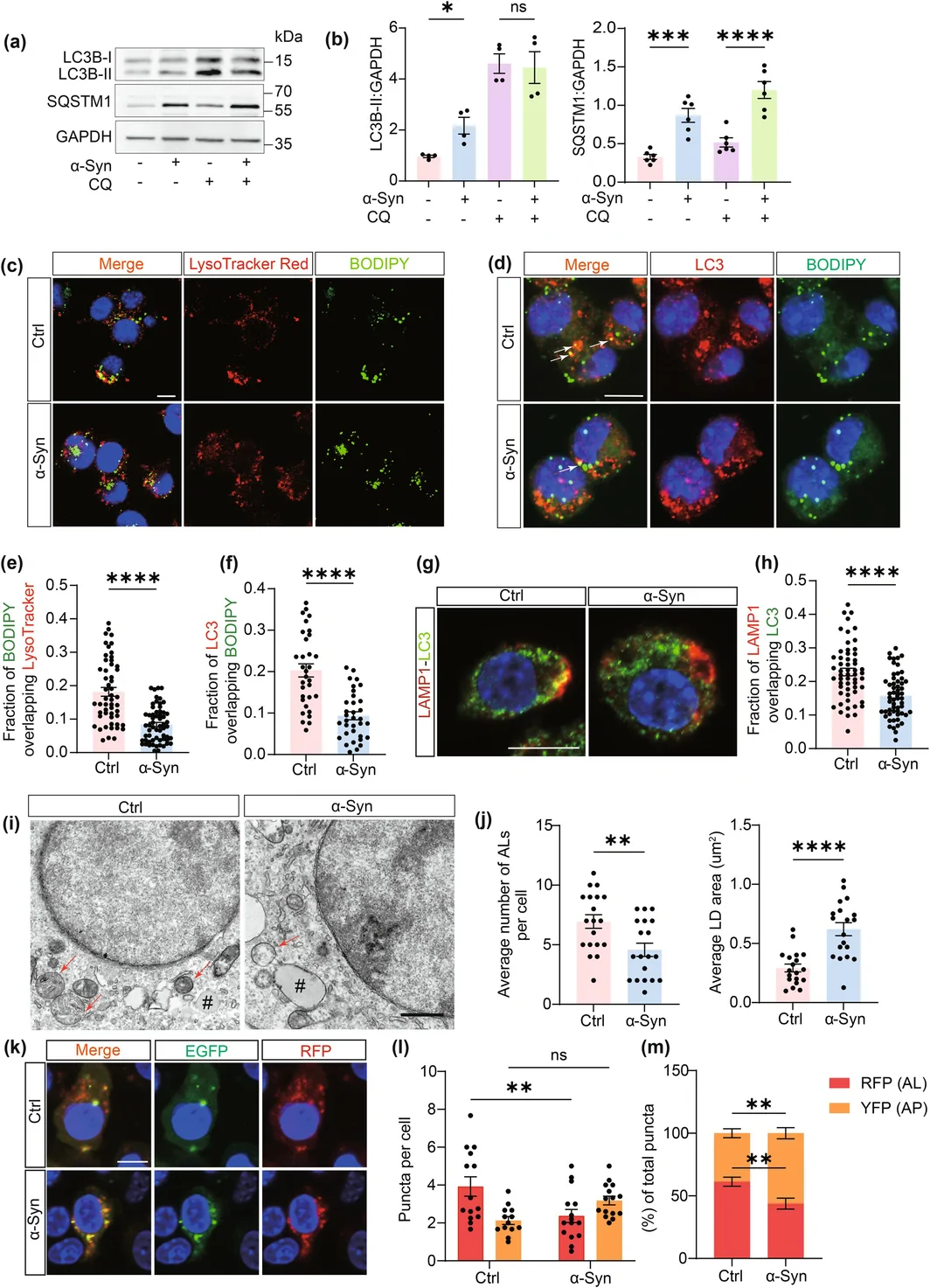
α‐Syn impairs lipophagy by suppressing autophagosome‐lysosome fusion. (a, b) Immunoblotting of autophagy markers LC3B‐II (*n* = 4) and SQSTM1 (*n* = 6) in RAW264.7 macrophages treated with or without α‐Syn in the presence or absence of CQ. (c–f) Representative confocal IF images and quantitative analysis showing colocalization between BODIPY and LysoTracker (c, e; *n* = 53–56) or LC3 (d, f; *n* = 32–34) in ox‐LDL alone or α‐Syn plus ox‐LDL treated RAW264.7. Scale bar: 10 μm. (g, h) Representative images showing colocalization between LC3 and LAMP1 in two treatment groups, along with quantitative analysis (*n* = 56–57). Scale bar: 10 μm. (i, j) Representative TEM images of AL and LDs in the two groups of treated cells, along with quantification of AL number and LDs area (*n* = 19). ^#^LDs, ALs (red arrows). Scale bar: 1 μm. (k–m) Autophagic flux analysis in cells expressing tandem RFP‐EGFP‐LC3 reporter: Representative images (k) and quantification showing the average number of red‐only puncta (RFP, AL) and yellow puncta (YFP, AP) in each cell (l), as well as the proportion of ALs and APs in the total number of dots (m) (*n* = 13–17). Scale bar: 10 μm. All data are shown as the mean ± SEM. Statistical analysis was performed using 2‐way ANOVA followed by Šídák's post hoc test (b, l, m), Welch's *t*‐test (f, right panel of j), Mann–Whitney *U* test (e, left panel of j), unpaired *t*‐test (h). ns, *p* > 0.05; *p < 0.05; **p < 0.01; ***p < 0.001; ****p < 0.0001.

Given that our previous experiments demonstrated that α‐Syn promoted macrophage foam cell formation, we next investigated whether this effect was associated with impaired autophagy. IF analysis revealed that after 24 h of α‐Syn treatment, the colocalization of LysoTracker, an acidic lysosomal probe, with BODIPY‐labeled lipid droplets was markedly reduced (Figure 4c,e). In parallel, the colocalization of LC3 with BODIPY was also decreased (Figure 4d,f). These findings indicate reduced interactions between LDs and both lysosomes and autophagosomes, suggesting impaired autophagy‐associated lipid processing in response to α‐Syn.

To conclusively validate whether α‐Syn impaired autophagy flux, we performed a series of complementary assays. First, IF staining was performed to assess the colocalization of LC3 (an autophagosome marker) with LAMP1 (lysosomal associated membrane protein 1, a lysosomal marker). A significant reduction of their colocalization in α‐Syn‐treated macrophages indicated impaired fusion between autophagosomes and lysosomes (Figure 4g,h). Second, transmission electron microscopy (TEM) was employed to visualize the ultrastructural features of autophagic compartments. We observed that compared with the control group, α‐Syn‐treated macrophages exhibited a reduced number of autolysosomal structures and larger LDs (Figure 4i,j). Finally, we established a macrophage cell line stably expressing the tandem fluorescent reporter RFP‐EGFP‐LC3 to monitor autophagic flux (Klionsky et al. 2016). In this reporter system, EGFP fluorescence is quenched in the acidic lysosomal environment, whereas RFP remains stable, allowing discrimination between autophagosomes (RFP^+^ EGFP^+^, yellow puncta) and autolysosomes (RFP^+^ EGFP^−^, red‐only puncta). Following α‐Syn treatment, although the number of yellow puncta showed no obvious change, red puncta were markedly reduced (Figure 4k,l). Quantitative analysis revealed a decreased proportion of red‐only puncta and an increased proportion of yellow puncta relative to total LC3 puncta compared with the control group (Figure 4k,m). It demonstrates that after the α‐Syn treatment, the autophagy process fails to complete and remains at the stage where the autophagosomes fuse with the lysosomes, ultimately resulting in the accumulation of LC3B‐II and SQSTM1.

Together with the above results, these findings suggest that α‐Syn inhibits autophagosome‐lysosome fusion, leading to increased macrophage foam cell formation.

### 2.4 PI3K‐AKT–mTOR‐S6K Signaling Activation Underlies α‐Syn‐Induced Autophagy Impairment

The fusion of autophagosomes with lysosomes is orchestrated by a specific set of the soluble N‐ethylmaleimide‐sensitive factor attachment protein receptor (SNARE) proteins, including synaptosome‐associated protein 29 (SNAP29), syntaxin 17 (STX17), and vesicle‐associated membrane protein 8 (VAMP8) (Ke 2024). WB analysis showed that the expression of these proteins was not altered by α‐Syn treatment (Figure 5a,b). We therefore investigated upstream regulatory pathways of autophagy. AMP‐activated protein kinase (AMPK) and mammalian target of rapamycin (mTOR) are canonical upstream regulators of autophagy initiation and progression (Liu and Sabatini 2020; Mihaylova and Shaw 2011). We found that α‐Syn treatment markedly increased the phosphorylation of mTOR and its downstream effector ribosomal protein S6 kinase (S6K), as well as unc‐51 like autophagy activating kinase 1 (ULK1) at Ser 757, whereas the phosphorylation of AMPK remained unchanged (Figure 5c–f). These results further indicate that the accumulation of LC3B‐II induced by α‐Syn is unlikely due to enhanced autophagy initiation, but rather associated with impaired autophagic flux. As phosphoinositide 3‐kinase (PI3K)‐protein kinase B (PKB/AKT) signaling acts as a key upstream activator of mTOR, we next assessed this pathway. α‐Syn treatment resulted in increased phosphorylation of both PI3K and AKT (Figure 5g,h). To further evaluate the involvement of PI3K in α‐Syn‐induced mTOR activation, macrophages were pretreated 1h with the PI3K inhibitor LY294002 before α‐Syn stimulation. LY294002 reduced the α‐Syn‐induced increase in mTOR phosphorylation (Figure 5i,j), supporting the involvement of the PI3K signaling in mTOR activation.

**FIGURE 5:**
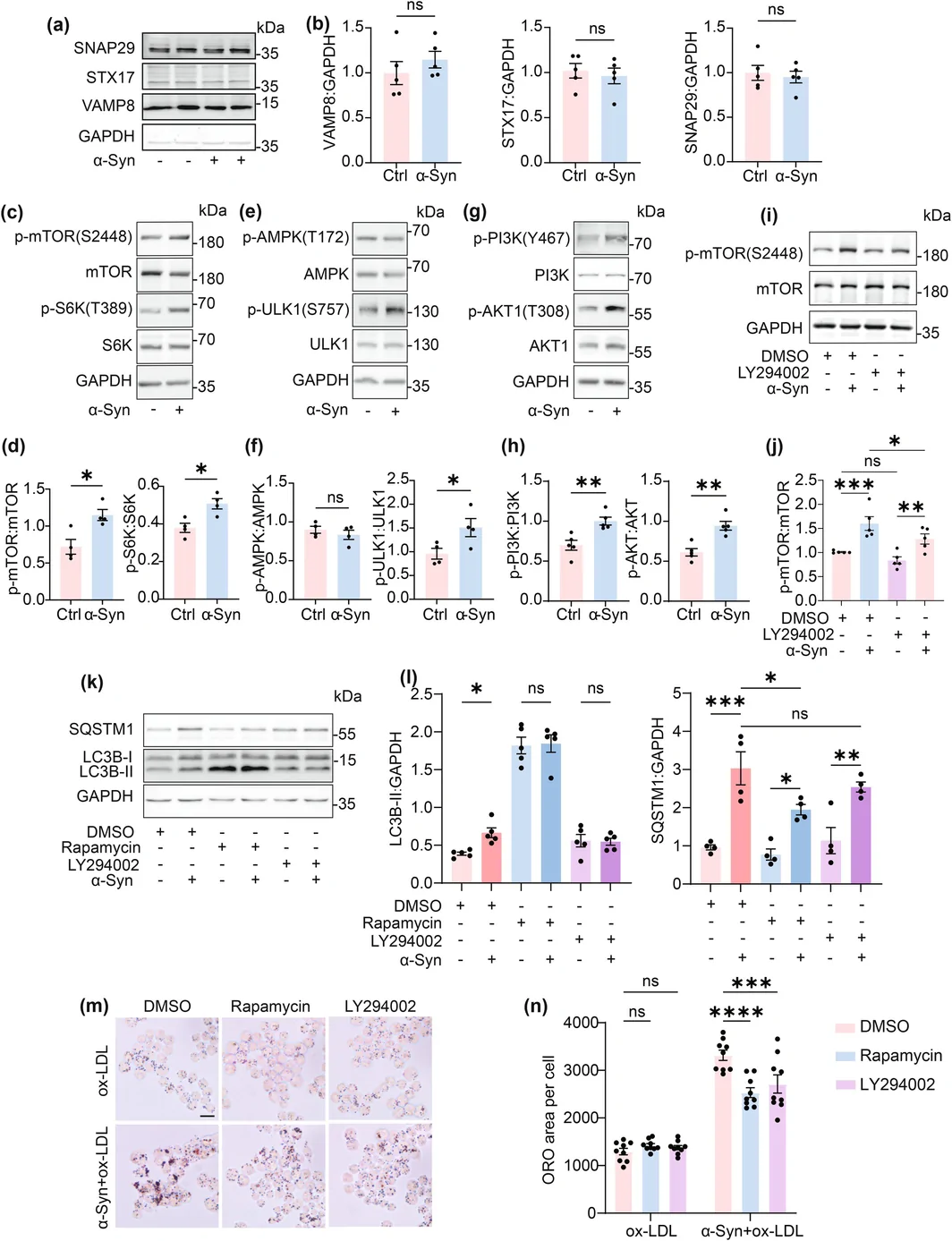
Activation of the PI3K‐AKT–mTOR‐S6K signaling mediates α‐Syn‐induced autophagic flux impairment and macrophage foam cell formation. (a, b) Representative western blots of key autophagosome‐lysosome fusion machinery proteins (SNAP29, STX17, and VAMP8) in RAW264.7 treated with ox‐LDL or α‐Syn plus ox‐LDL. Quantification of protein levels was normalized to GAPDH (*n* = 5). (c, d) Representative western blots and the normalized ratios of *p*‐mTOR:MTOR, *p*‐S6K:S6K in RAW264.7 treated with or without α‐Syn (*n* = 4). (e, f) Representative western blots and the normalized ratios of *p*‐AMPK:AMPK and p‐ULK1:ULK1 in RAW264.7 treated with or without α‐Syn (*n* = 4). (g, h) Representative western blots and the normalized ratios of p‐PI3K:PI3K, p‐AKT1:AKT1 in RAW264.7 treated with or without α‐Syn (*n* = 5). (i, j) Macrophages were pretreated for 1 h with LY294002 (PI3K inhibitor)or DMSO before α‐Syn exposure. Representative immunoblots and quantification of the p‐mTOR:MTOR (*n* = 5). (k, l) Macrophages were pretreated for 1 h with rapamycin (mTOR inhibitor) or LY294002, with DMSO before α‐Syn exposure. Representative western blots and quantification of LC3B‐II (*n* = 5) and SQSTM1 (*n* = 4). (m, n) Representative ORO staining and quantification of lipid accumulation in macrophages after 1 h pretreatment with rapamycin, LY294002, or DMSO, followed by exposure to ox‐LDL alone or α‐Syn plus ox‐LDL (*n* = 9). Scale bars: 20 μm. All data are shown as the mean ± SEM. Statistical analysis was performed using unpaired *t*‐test (b, d, f, h) and 2‐way ANOVA followed by Šídák's post hoc test (j, l, n). ns, *p* > 0.05; *p < 0.05; **p < 0.01; ***p < 0.001; ****p < 0.0001.

To determine whether inhibition of PI3K‐mTOR signaling alleviates α‐Syn‐induced autophagic dysfunction, cells were pretreated 1 h with rapamycin, a mTOR inhibitor, or LY294002 prior to α‐Syn exposure. Under the conditions of the two inhibitors, the α‐Syn‐induced increase in LC3B‐II levels was no longer observed (Figure 5k,l). Although SQSTM1 levels remained elevated, rapamycin pretreatment partially reduced SQSTM1 accumulation in α‐Syn‐treated cells compared with the corresponding groups without inhibitor pretreatment (Figure 5k,l), which may be attributable to increased SQSTM1 synthesis, consistent with the previously observed upregulation of *Sqstm1* mRNA. These findings suggest that inhibition of mTOR signaling partially rescues α‐Syn‐induced impairment of SQSTM1 degradation. Furthermore, we examined whether inhibition of the PI3K‐mTOR pathway affects α‐Syn‐induced macrophage foam cell formation. ORO staining demonstrated that pretreatment with rapamycin or LY294002 attenuated α‐Syn‐induced lipid accumulation (Figure 5m,n), indicating that blockade of this pathway partially reverses the pro‐foam cell effect of α‐Syn.

Together, these findings suggest that activation of the PI3K‐AKT‐mTOR‐S6K signaling is associated with α‐Syn‐induced autophagic flux impairment and macrophage foam cell formation.

### 2.5 FLOT1 Mediates α‐Syn Internalization and Contributes to α‐Syn‐Induced Autophagy Impairment and Lipid Accumulation

Although α‐Syn activated the PI3K‐AKT‐mTOR‐S6K signaling, the upstream mechanism remains unclear. To identify potential membrane‐associated mediators, we looked for clues in the proteomics between RAW264.7 treated with ox‐LDL alone or α‐Syn plus ox‐LDL. Among the differentially expressed proteins, flotillin‐1 (FLOT1) was markedly upregulated in α‐Syn‐treated cells (Figure S8). WB analysis further validated the proteomic findings, demonstrating that treatment with α‐Syn increased the protein levels of FLOT1 (Figure 6a).

**FIGURE 6:**
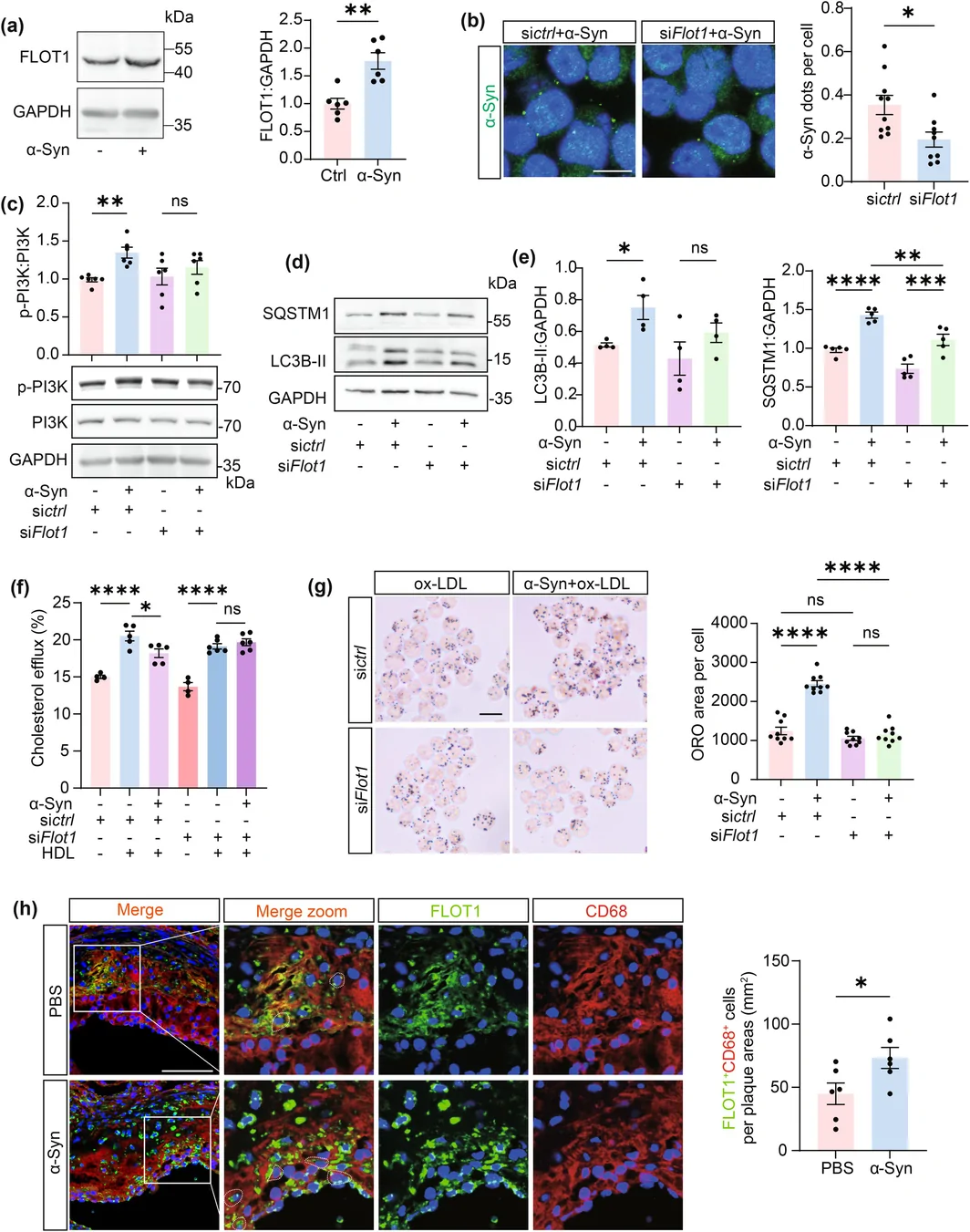
FLOT1 facilitates α‐Syn internalization and contributes to α‐Syn‐induced autophagy impairment. (a) Immunoblot validation of increased FLOT1 expression in RAW264.7 cells following α‐Syn plus ox‐LDL treatment (*n* = 6). (b) Representative IF images showing intracellular α‐Syn dots and quantification of α‐Syn endocytosis after 1‐h treatment in si*ctrl* versus si*Flot1* cells (*n* = 9–10). Scale bars: 10 μm. (c) Representative western blots and the normalized ratios of p‐PI3K:PI3K in RAW264.7 treated with or without α‐Syn (*n* = 6). (d, e) Representative western blots (d) and quantification (e) of LC3B‐II (*n* = 4) and SQSTM1 (*n* = 5) in si*ctrl* and si*Flot1* cells treated with ox‐LDL or α‐Syn plus ox‐LDL. (f) HDL‐mediated cholesterol efflux was measured in macrophages transfected with a control siRNA (si*ctrl*) or an *Flot1* siRNA (si*Flot1*) (*n* = 4–6). (g) ORO staining and quantification of intracellular ORO positive area in si*ctrl* and si*Flot1* cells treated with ox‐LDL or α‐Syn plus ox‐LDL (*n* = 9). Scale bars: 20 μm. (h) Representative IF images of aortic root plaques stained for FLOT1 and CD68 with quantification of the density of FLOT1^+^ CD68^+^ cells per mm^2^ plaque area in *Apoe* ^−/−^ mice injected with PBS or α‐Syn (*n* = 6). Scale bars: 100 μm. All data are shown as the mean ± SEM. Statistical analysis was performed using unpaired *t*‐test (a, b, h), 2‐way ANOVA followed by Šídák's post hoc test (c, e, g), and 1‐way ANOVA followed by Šídák's post hoc test (f). ns, *p* > 0.05; *p < 0.05; **p < 0.01; ***p < 0.001; ****p < 0.0001.

FLOT1 is a membrane raft‐associated protein that has been reported to participate in α‐Syn endocytosis in previous studies (Kobayashi et al. 2019). To elucidate the involvement of FLOT1 in α‐Syn‐mediated foam cell formation, RAW264.7 macrophages were transfected with *Flot1*‐specific siRNA (si*Flot1*), while cells transfected with a non‐targeting siRNA (si*ctrl*) were used as controls (Figure S9). Compared with si*ctrl*‐transfected cells, *Flot1* knockdown significantly decreased the endocytosis of α‐Syn after 1 h of treatment (Figure 6b), suggesting that FLOT1 contributes to α‐Syn internalization in macrophages. To further characterize the association between FLOT1 and α‐Syn, we performed additional IF colocalization and co‐immunoprecipitation (Co‐IP) assays to assess their intracellular spatial proximity and biochemical association. RAW264.7 macrophages were incubated with 200 nM exogenous α‐Syn for 1 h, followed by fixation and IF staining for FLOT1 and α‐Syn. Confocal microscopy revealed an evident spatial colocalization between intracellular α‐Syn and FLOT1 (Figure S10a). Co‐IP analysis further showed that GST‐tagged α‐Syn was detected in FLOT1‐Myc immunoprecipitates from co‐transfected HEK293 cells, but not in the empty‐vector control group (Figure S10b), supporting a protein‐level association between FLOT1 and α‐Syn. Collectively, these results indicate that FLOT1 facilitates the cellular uptake of α‐Syn and may serve as a membrane‐associated platform involved in α‐Syn internalization in macrophages.

In the oncologic context, upregulation of FLOT1 has been linked to activation of multiple signaling pathways, including PI3K‐AKT (Zhan et al. 2023). We therefore hypothesized that *Flot1* knockdown may restore autophagic flux by attenuating PI3K phosphorylation. Under si*ctrl* conditions, α‐Syn increased PI3K phosphorylation, whereas under si*Flot1* conditions, this response was abolished (Figure 6c). Similarly, upon *Flot1* knockdown, α‐Syn no longer induced LC3B‐II accumulation, and the extent of SQSTM1 upregulation was reduced (Figure 6d,e). These findings indicate that *Flot1* knockdown alleviates the α‐Syn‐induced blockade of autophagic flux, thereby partially restoring autophagy.

One functional consequence of restored autophagic flux is increased autophagy‐dependent lipid degradation. We first determine whether *Flot1* knockdown altered the effect of α‐Syn on cholesterol efflux. Under si*ctrl* conditions, α‐Syn inhibited HDL‐mediated cholesterol efflux, while under si*Flot1* conditions, α‐Syn no longer suppressed HDL‐mediated cholesterol efflux (Figure 6f). Subsequently, both si*ctrl* and si*Flot1* RAW264.7 were subjected to in vitro foam cell induction in the presence or absence of α‐Syn. The ORO staining showed that knockdown of *Flot1* attenuated α‐Syn‐induced macrophage foam cell formation (Figure 6g). Consistent with in vitro findings, we observed a higher density of FLOT1‐positive macrophages (FLOT1^+^ CD68^+^ cells) in aortic root plaques from α‐Syn‐injected *Apoe* ^−/−^ mice compared with the PBS‐treated group (Figure 6h). These findings indicate that *Flot1* knockdown prevents the α‐Syn‐induced reduction in macrophage cholesterol efflux and the consequent increase in foam cell formation.

Together, these results identify FLOT1 as an upstream contributor to α‐Syn‐induced PI3K activation, autophagy dysregulation, and lipid accumulation.

## 3 Discussion

This study investigated whether α‐Syn contributes to atherosclerosis progression and explored the underlying mechanisms by combining evidence from human lesions, in vivo models, and in vitro functional assays.

α‐Syn is present not only in the brain but also in peripheral tissues, through either local expression (Burre et al. 2018) or CNS‐derived transport (Gilboa et al. 2024; Shi et al. 2014). Prior studies have demonstrated α‐Syn expression in human cerebrovascular walls (Tamo et al. 2002), and its secretion by human‐derived endothelial cells (Takami et al. 2022). Complementing these findings, we detected α‐Syn‐specific peptides by LC‐MS/MS and multiple α‐Syn‐immunoreactive bands by WB in human atherosclerotic plaque tissue. Beyond its local presence, α‐Syn has been implicated in circulatory disorders. In addition, an immune atlas of human atherosclerosis linked SNCA/α‐Syn to plaque immune regulation (Barcia Duran et al. 2024), and a stroke model showed that cerebral ischemia induced pathological α‐Syn alterations and promoted endothelial inflammation (Bogale et al. 2025). Together with epidemiological data showing an elevated stroke risk in PD patients (Park et al. 2020), these observations support a potential role for α‐Syn in vascular pathology.

Using *Snca*‐deficient mice, we observed a consistent atheroprotective phenotype, characterized by reduced macrophage‐associated lipid accumulation. Previous studies of *Snca* knockout mice focused mainly on neuronal development (Garcia‐Reitboeck et al. 2013) or cognitive function (Kokhan et al. 2012), with some evidence suggesting a role in vascular contractility (Marrachelli et al. 2010). Our study focuses on the progression of atherosclerosis in the peripheral circulatory system, which provides a significant contribution to the current body of knowledge. Future investigations may employ more advanced methodologies to further explore the impact of α‐Syn on cerebrovascular health.

Exogenous α‐Syn exposure enhanced macrophage foam cell formation and accelerated atherosclerosis progression in vivo. Although α‐Syn is expressed in macrophages, its abundance is extremely low (Grozdanov and Danzer 2020; Pei and Maitta 2019); thus, loss of endogenous α‐Syn expression alone may not materially affect macrophage lipid metabolic capacity. Moreover, the cellular α‐Syn pool derives from both intrinsic expression and extracellular uptake (Limanaqi et al. 2024; Scheiblich et al. 2021). This raises the possibility that the atheroprotective phenotype observed in the *Snca* ^−/−^ mice may result from reduced exposure to exogenous (intravascular) α‐Syn, a hypothesis supported by our subsequent experiments. Purified α‐Syn significantly increased neutral LD accumulation in macrophages and promoted lesion development in *Apoe* ^−/−^ mice. These observations imply that exogenous α‐Syn contributes to atherosclerosis progression.

Mechanistically, our data indicated that α‐Syn inhibited autophagy by impairing autophagosome‐lysosome fusion, accompanied by activation of the PI3K‐AKT‐mTOR axis. Both cellular and in vivo analyses demonstrated impaired autophagic flux. SQSTM1 accumulation was partly due to the transcriptional upregulation, which may additionally reflect inflammatory signaling, as NF‐κB can activate *Sqstm1* transcription (Huang et al. 2016; Zhong et al. 2016). Regarding the fusion defect, a previous study reported that α‐Syn overexpression impaired autophagosome‐lysosome fusion in neurons by binding SNAP29 and promoting its degradation (Tang et al. 2021). However, we observed no reduced expression of the STX17‐SNAP29‐VAMP8 complex in macrophages, suggesting cell‐type specificity. Instead, α‐Syn increased phosphorylation of PI3K and AKT, accompanied by activation of the mTOR‐S6K‐ULK1 cascade. The PI3K‐AKT‐mTOR signaling is a central growth‐metabolic signaling axis that integrates extracellular growth cues and intracellular energy status to regulate autophagy (Fruman et al. 2017). Treatment with the PI3K inhibitor LY294002 or the mTOR inhibitor rapamycin partially normalized α‐Syn‐induced LC3B‐II abnormalities and reduced lipid droplet accumulation, implicating the PI3K‐mTOR axis in α‐Syn‐mediated autophagic blockade and foam cell formation.

Previous studies indicate the cellular uptake of α‐Syn and the subsequent activation of downstream signaling pathways, although this likely depends on cellular context and α‐Syn conformational state (Dorion et al. 2024; Kim et al. 2013; Mao et al. 2016; Wu et al. 2025). Our results support a FLOT1‐related entry route for α‐Syn in macrophages, consistent with previously reported receptors mediating α‐Syn uptake in dopaminergic neurons (Kobayashi et al. 2019). IF colocalization and Co‐IP supported spatial and biochemical association between FLOT1 and α‐Syn, although these assays do not establish direct binding. Mechanistically, eukaryotic translation initiation factor 4A3 (EIF4A3) has been shown to regulate the PI3K‐AKT‐ERK1/2‐P70S6K pathway via FLOT1 in lung adenocarcinoma (Yu et al. 2023). In our study, *Flot1* knockdown attenuated α‐Syn‐induced PI3K phosphorylation, relieved autophagic flux blockade, and blunted foam cell augmentation. These findings identify FLOT1 as a candidate membrane‐associated mediator linking extracellular α‐Syn to PI3K‐mTOR signaling in macrophages.

In conclusion, this study reveals that FLOT1 facilitates α‐Syn uptake in macrophages and that extracellular α‐Syn impairs autophagosome‐lysosome fusion, promotes macrophage foam cell formation, and ultimately accelerates atherosclerosis progression. Pharmacological inhibition of PI3K or mTOR, or knockdown of *Flot1*, partially restores α‐Syn‐induced macrophage autophagy suppression and lipid accumulation. These findings identify FLOT1 and the PI3K‐mTOR axis as potential modulators of α‐Syn‐associated vascular pathology in atherosclerosis, raising the possibility of therapeutic targeting, particularly in aging populations where α‐Syn pathology and vascular disease may converge.

## 4 Methods

### 4.1 Human Tissue Samples

Human atherosclerotic plaque specimens (*n* = 3) were collected from patients undergoing carotid endarterectomy. The patients had symptomatic or asymptomatic carotid artery stenosis of ≥ 70% and met the clinical indications for carotid endarterectomy. The study protocol was reviewed and approved by the Ethics Committee of Union Hospital, Tongji Medical College, Huazhong University of Science and Technology, Wuhan, China (approval no. [2025]‐1284‐01). Written informed consent was obtained from all participants prior to sample collection.

### 4.2 Regents and Antibodies

The following reagents were used: NBD‐cholesterol (Biofount, 78949‐95‐8; 1 μg/mL), CQ (Sigma‐Aldrich, C6628; 50 μmol/L), LY294002 (MedChemExpress, HY‐10108; 10 μmol/L), rapamycin (MedChemExpress, HY‐10219; 100 nmol/L), ox‐LDL (Yiyuan Biotech, YB‐002; 50 μg/mL), DiI‐ox‐LDL (Yiyuan Biotech, YB‐0010; 10 μg/mL), APOA1 (Novaprotein, P02647; 10 μg/mL), and HDL (Yiyuan Biotech, YB‐003; 50 μg/mL). The following primary antibodies for immunofluorescence were used: anti‐LAMP1 (Proteintech, 67300‐1‐Ig), anti‐FLOT1 (Proteintech, 15571‐1‐AP), anti‐CD68 (Servicebio, GB113109), anti‐CD68 (Servicebio, GB14043), anti‐ACTA2 (Servicebio, GB13044), anti‐LC3 (Medical & Biological Laboratories Co, PM036), and anti‐SQSTM1 (Cell Signaling Technology, 23214). The following secondary antibodies were used for western blot experiments: anti‐MSR1 (Absin, abs136214), anti‐CD36 (Proteintech, 18836‐1‐AP), anti‐OLR1 (ABclonal, A1639), anti‐ABCA1 (Abcam, ab18180), anti‐ABCG1 (ABclonal, A17907), anti‐LC3B‐II (Proteintech, 14600‐1‐AP), anti‐SQSTM1 (Cell Signaling Technology, 5114), anti‐p‐mTOR‐S2448 (Cell Signaling Technology, 5536), anti‐mTOR (Cell Signaling Technology, 2983), anti‐p‐p70 S6K‐T389 (Cell Signaling Technology, 9234), anti‐p70 S6K (Cell Signaling Technology, 2708), anti‐p‐AMPK1‐T172 (Cell Signaling Technology, 2535), anti‐AMPK1 (Zen‐bioscience, R380431), anti‐p‐ULK1‐S757 (Cell Signaling Technology, 14202), anti‐ULK1 (abmart, T56902F), anti‐p‐PI3K p85‐Y467 (Zen‐bioscience, R341468), anti‐PI3K antibody (Proteintech, 60225‐1‐Ig), anti‐p‐AKT1‐T308 (Cell Signaling Technology, 13038), anti‐AKT1 (Cell Signaling Technology, 4691), anti‐α‐Synuclein (ABclonal, A22414 and A27116), anti‐α‐Synuclein (clone Syn211, Thermo Fisher Scientific, MA5‐12272), anti‐FLOT1 (Proteintech, 15571‐1‐AP), anti‐GST (Beyotime Biotechnology, AF2299), anti‐Myc (Proteintech, 10828‐1‐AP), anti‐GAPDH (ABclonal, A19056), and anti‐β‐tubulin (Servicebio, GB15140). The following secondary antibodies were used for western blot experiments: anti‐rabbit IgG light chain (Abbkine, A25022) HRP‐conjugated secondary antibody, anti‐mouse (ABclonal, AS003), and anti‐rabbit (ABclonal, AS014) HRP‐conjugated IgGs. The following secondary antibodies were used for immunofluorescence experiments: anti‐mouse Alexa Fluor 488‐conjugated (Thermo Fisher Scientific, R37114), anti‐mouse Alexa Fluor 594‐conjugated (Thermo Fisher Scientific, R37115), anti‐rabbit Alexa Fluor 488‐conjugated (Thermo Fisher Scientific, R37118), anti‐rabbit IgG (H + L) cross‐adsorbed Alexa Fluor 594 (Thermo Fisher Scientific, A‐21207).

### 4.3 Preparation of α‐Syn Monomer

cDNAs corresponding to His‐tagged mouse‐α‐Syn or His‐tagged human‐α‐Syn were cloned into a pRK172 plasmid and transformed into the *E. coli* BL21 (DE3) strain. The expression and purification were performed as previously described (Volpicelli‐Daley et al. 2014). Briefly, the pellet from 1‐L culture was resuspended in 100 mL osmotic shock buffer (30 mM Tris–HCl, 40% sucrose, and 2 mM EDTA, pH 7.2) and incubated for 10 min at room temperature (RT). The pellet collected by centrifugation at 12,000 rpm for 20 min was resuspended quickly with 90 mL cold water followed by adding 37.5 μL of saturated MgCl_2_ and kept on ice for 3 min. His‐tagged proteins were purified through Ni‐chelating affinity chromatography and eluted at around 125 mM imidazole. The protein was dialyzed, lyophilized, and stored at −80°C before use. The recombinant α‐Syn used in this study had an endotoxin level of < 0.1 EU/mg, as determined by ToxinSensor Chromogenic LAL Endotoxin Assay Kit (L00350C).

### 4.4 Animals

*Snca* knockout (*Snca* ^−/−^) mice were from Jackson Laboratory (stock number: 003692). To generate experimental animals, these mice were crossed with wild‐type mice. The resulting heterozygous offspring were then intercrossed to produce *Snca* ^−/−^ mice and their littermate wild‐type controls, and both male and female mice were used. Male *Apoe* knockout (*Apoe* ^−/−^) mice on a C57BL/6J background were obtained from Vital River Laboratory Animal Technology Co. Ltd. (Beijing, China). All mice were maintained under specific pathogen‐free conditions with controlled temperature and humidity on a 12‐h light/dark cycle, with ad libitum access to food and water. All animal procedures were approved by the Institutional Animal Care and Use Committee of the corresponding institution and were performed in accordance with relevant guidelines (IACUC5078).

To induce atherosclerosis, 8‐week‐old *Snca* ^−/−^ mice and *Snca* ^+/+^ littermates received a single tail‐vein injection of AAV‐PCSK9 (200 μL per mouse, 1.0 × 10^12^ viral genome titer per mouse) which was diluted in sterile PBS. Following 1 week after AAV‐PCSK9 administration, mice were fed a HCD (#XT108C, Xietong Shengwu, Nanjing, China) for 16 weeks.

To evaluate the pro‐atherogenic effects of α‐Syn in vivo, 8‐week‐old male *Apoe* ^−/−^ mice with comparable body weight were randomly assigned to receive α‐Syn or PBS. Mice were administered mouse‐derived monomeric α‐Syn (50 μg per mouse, dissolved in sterile PBS) by tail‐vein injection. The injection dose of α‐Syn is based on literature exploring the induction of CNS lesions by peripheral injection of α‐Syn fibrils (Ayers et al. 2017). As the half‐life of endogenous α‐Syn is 16.8 ± 2 h (Cuervo et al. 2004), we adopt an injection frequency of twice a week. For each injection, α‐Syn was diluted with sterile normal saline to a final volume of 200 μL. Injections were performed twice weekly throughout 12 weeks of HCD feeding.

At the endpoint, mice were anesthetized and perfused with PBS. The entire aorta, including the aortic arch and major branches, was harvested for en face ORO staining to quantify atherosclerotic lesion burden. Hearts containing the aortic root were embedded and cryosectioned for histological and immunostaining analyses. Tissues were processed and stored appropriately (fixed or snap‐frozen at −80°C) according to downstream assays.

### 4.5 Atherosclerotic Lesion Analysis

The entire aorta (from the aortic root to the iliac bifurcation) was carefully dissected and collected for en face ORO staining. Hearts were excised and fixed in 4% paraformaldehyde (PFA) overnight, then embedded for cryosectioning. Serial cryosections of the aortic root were cut at 6 μm thickness, collected onto slides, and stored at −80°C until use for histological staining and immunofluorescence analyses.

### 4.6 Cell Culture and Transfection

RAW264.7 macrophages were obtained from Cyagen (Oricell, M3‐0101) and maintained in Dulbecco's modified Eagle's medium (DMEM) (Servicebio, G4511) supplemented with 10% fetal bovine serum and 1% penicillin–streptomycin. Cells were cultured at 37°C in a humidified incubator with 5% CO_2_ and passaged every 2 days to maintain exponential growth.

For the genetic knockdown of *Flot1*, RAW264.7 macrophages were seeded in culture plates and allowed to adhere overnight. On the next day, cells reached approximately 50%–70% confluence and were transfected with *Flot1*‐specific siRNA siRNA (si*Flot1*) or non‐targeting control siRNA (si*ctrl*) using a commercial transfection reagent [CALNP RNAi in vitro transfection reagent (DN001‐10, Beijing, China)] according to the manufacturer's instructions. SiRNAs were used at 200 nM. Knockdown efficiency was determined by western blotting at 24 h post‐transfection, prior to subsequent experiments. The siRNAs were synthesized by Wuhan AuGCT DNA‐SYN Biotechnology Co. Ltd. The sequences of the siRNAs against *Flot1* were as follows: sense, 5′‐CCUACGACAUCGAGGUCAACATT‐3′; anti‐sense, 5′‐UUGACCUCGAUGUCGUAGGUGTT‐3′. The sequences of the non‐targeting control siRNAs were as follows: sense, 5′‐UUCUCCGAACGUGUCACGUTT‐3′; anti‐sense, 5′‐ACGUGACACGUUCGGAGAATT‐3′.

### 4.7 BMDM Isolation and Differentiation

BMDMs were isolated as previously described (Cui et al. 2024). Briefly, bone marrow cells were flushed from femurs and tibias, passed through a 70‐μm strainer, pelleted, and treated with red blood cell lysis buffer (Servicebio, G2015). After washing, cells were resuspended in RPMI‐1640 containing 10% fetal bovine serum, 1% penicillin–streptomycin, and 20 ng/mL macrophage colony‐stimulating factor, and plated. Cultures were maintained for 5–7 days with 50% medium changes every 2 days, yielding adherent BMDMs for downstream assays.

### 4.8 ORO Staining

ORO staining was used to detect neutral lipids. For tissues, 6‐μm frozen sections were air‐dried, rinsed, equilibrated in 60% isopropanol, and incubated with ORO working solution (freshly diluted from isopropanol stock) for 10–15 min at RT, followed by rinsing in 60% isopropanol and water, then mounted and imaged. For cells, macrophages were fixed in 4% PFA, rinsed, and stained with ORO as above, then washed and imaged. ORO‐positive area or integrated optical density was quantified using ImageJ and normalized to total section area or cell number.

### 4.9 BODIPY Staining

LDs in cells or tissue sections were stained with BODIPY (Thermo Fisher Scientific, D3922) for 30 min, followed by processing for immunofluorescence as previously described (Jeong et al. 2018). Staining was assessed by Olympus IX73 fluorescence microscopy (Olympus, Japan) or Nikon A1‐Si confocal microscopy (Nikon, Tokyo, Japan). Quantification was performed using ImageJ software after appropriate thresholding.

### 4.10 Analysis of DiI‐oxLDL Uptake

Cells were seeded and allowed to adhere overnight, then incubated with 10 μg/mL DiI‐labeled ox‐LDL (DiI‐oxLDL) (Yiyuan Biotech, YB‐0010) in complete medium for 4 h. After incubation, cells were washed three times with PBS, fixed with 4% PFA for 10 min, and washed again. Nuclei were counterstained with DAPI. Fluorescence images were acquired, and the mean fluorescence intensity (mFI) was quantified using ImageJ.

### 4.11 Analysis of Cholesterol Efflux

The cholesterol efflux assay was described before (Pi et al. 2021). Briefly, cells were seeded in plates and then incubated with 5 μM 22‐NBD‐cholesterol to label intracellular cholesterol pools for 4 h. After labelling, cells were washed gently with PBS to remove unincorporated probe and equilibrated in serum‐free medium for a short period. Cholesterol efflux was initiated by adding medium containing 50 μg/mL HDL or 10 μg/mL APOA1 and incubating the cells for 4 h at 37°C. At the end of the efflux period, culture supernatants were collected, and cells were lysed using 0.3 M NaOH solution. Fluorescence intensity of 22‐NBD‐cholesterol in the supernatants and corresponding cell lysates was measured using a multimode microplate reader at excitation/emission wavelengths of 472/570 nm. The cholesterol efflux rate was calculated as: efflux (%) = fluorescence in supernatant/(fluorescence in supernatant + fluorescence in cell lysate) × 100, after background subtraction.

### 4.12 LysoTracker Red Staining

Cells were incubated with pre‐warmed LysoTracker Red (Beyotime Biotechnology, C1046) working solution for 60 min at 37°C, then washed three times with PBS and fixed with 4% PFA for 10 min. After fixation, cells were washed again and stained with 200 μL of Hoechst 33342 (Beyotime Biotechnology, C1025) for 30 min at RT, followed by three PBS washes. Fluorescent signals were imaged using a Nikon A1‐Si confocal microscope.

### 4.13 Immunofluorescence Staining

For immunofluorescence staining, cells were fixed with 4% PFA for 10–15 min, whereas 6‐μm aortic root cryosections were equilibrated to RT for 30 min. Samples were permeabilized with 0.3% Triton X‐100 for 10 min, blocked with 5% BSA for 30 min at RT, and incubated with primary antibodies overnight at 4°C. After PBS washes, samples were incubated with fluorophore‐conjugated secondary antibodies for 1 h at RT in the dark, followed by DAPI counterstaining for 5–10 min. Images were acquired using a Nikon AXR NSPARC confocal microscope. For quantitative analysis of mouse aortic root sections, two sections from each mouse were analyzed, and the values obtained from the two sections were averaged to yield one biological replicate per mouse. Colocalization analysis of culture cells was performed using ImageJ with the JACoP plugin. Manders' coefficients were calculated to quantify the fraction of fluorescence in each channel overlapping with the other channel.

### 4.14 RFP‐EGFP‐LC3 Reporter

To monitor autophagic flux, we utilized a RAW264.7‐derived stable cell line expressing the RFP‐EGFP‐LC3 reporter, generated by Corues Biotechnology (Nanjing, China). After the indicated treatments, cells were fixed with 4% PFA, counterstained with DAPI, and mounted with an anti‐fade mounting medium. Images were acquired using a confocal microscope with a 60× objective under identical settings across groups. Autophagosomes (yellow puncta; RFP^+^ EGFP^+^) and autolysosomes (red‐only puncta; RFP^+^ EGFP^−^) were quantified using ImageJ. For each field, red‐only and yellow puncta were counted and normalized to the average cell number per field, and the ratio of red‐only to all puncta was calculated as an index of autophagic flux.

### 4.15 Electron Microscopy

The cells were collected after the indicated treatments and immediately fixed in 2.5% glutaraldehyde (in 0.1 M phosphate buffer, pH 7.4) at 4°C, followed by post‐fixation in 1% osmium tetroxide. Samples were then dehydrated through a graded ethanol series and embedded in epoxy resin. Ultrathin sections (~70 nm) were cut using an ultramicrotome, mounted on copper grids, and stained with uranyl acetate and lead citrate. Sections were examined with a transmission electron microscope to evaluate autolysosomes, based on established ultrastructural criteria (Klionsky et al. 2021).

### 4.16 Western Blot Analysis

Cells were washed with PBS and lysed in RIPA buffer supplemented with protease and phosphatase inhibitor cocktails on ice. Approximately 15 mg of frozen (−80°C) human plaque tissue was minced with sterile scissors and homogenized in 150 μL of ice‐cold RIPA lysis buffer containing a protease inhibitor cocktail, with all procedures performed on ice. The homogenate and lysates were clarified by centrifugation, and protein concentrations were determined using a BCA assay. Equal amounts of protein were mixed with SDS sample loading buffer, boiled, separated by SDS‐PAGE, and transferred onto PVDF membranes. Membranes were blocked with 5% non‐fat milk or 5% BSA in TBST for 1 h at RT and incubated with primary antibodies overnight at 4°C. After washing with TBST, membranes were incubated with HRP‐conjugated secondary antibodies for 1 h at RT. Immunoreactive bands were visualized using enhanced chemiluminescence and imaged with a chemiluminescence detection system. Band intensities were quantified using ImageJ, normalized to the indicated loading control (β‐tubulin or GAPDH), and expressed relative to the control group.

### 4.17 Reverse Transcription qPCR

Total RNA was extracted from cells using Trizol reagent (Invitrogen, 15596026). RNA concentration and purity were assessed by spectrophotometry, and equal amounts of RNA were reverse‐transcribed into cDNA using the HiScripty IV RT SuperMix Kit (Vazyme, R423‐01). Quantitative PCR was performed using the SYBR Premix Ex TaqTM Kit (Takara, RR420A) on a thermocycler (Bio‐Rad, Hercules, CA, USA). Gene‐specific primers for *Lc3b* and *Sqstm1* were used, with *Gapdh* as an internal control. The primers for *Lc3b* were 5′‐TTATAGAGCGATACAAGGGGGAG‐3′ (forward) and 5′‐CGCCGTCTGATTATCTTGATGAG‐3′ (reverse). The primers for *Sqstm1* were 5′‐ATGTGGAACATGGAGGGAAGA‐3′ (forward) and 5′‐GGAGTTCACCTGTAGATGGGT‐3′ (reverse). The primers for Gapdh were 5′‐AGCCTGCGGCTTAATTTGAC‐3′ (forward) and 5′‐CAACTAAGAACGGCCATGCA‐3′ (reverse). Relative mRNA expression levels were calculated using the 2^−ΔΔCt^ method and are presented as fold change compared with the control group (Livak and Schmittgen 2001). All RT‐qPCR experiments were performed with at least three independent biological replicates.

### 4.18 LC‐MS/MS

Human carotid plaque proteins were extracted, digested with trypsin, and analyzed by dia‐PASEF LC‐MS/MS using a timsTOF HT mass spectrometer coupled to a Vanquish Neo liquid chromatography system. Raw DIA data were processed using the library‐free directDIA workflow in Spectronaut (version 19) against the UniProtKB *Homo sapiens* reference proteome database, with peptide‐ and protein‐level false discovery rates controlled at 1%. For α‐synuclein‐specific peptides, precursor‐level, chromatographic, fragment‐ion, and mass‐accuracy information was manually inspected to support peptide identification.

For cellular proteomic analysis, proteins extracted from RAW264.7 cells after the indicated treatments were digested with trypsin and analyzed in dia‐PASEF mode using an UltiMate 3000 RSLCnano system coupled to a timsTOF Pro mass spectrometer. Raw data were processed using DIA‐NN software (version 1.8.1) in library‐free mode against the UniProtKB *Mus musculus* reference proteome database, with precursor‐ and protein‐level false discovery rates controlled at 1%. Proteins with at least 50% valid quantitative values in at least one group were retained. Differential protein abundance was assessed using Student's *t*‐tests followed by Benjamini–Hochberg correction, and proteins with an adjusted *p* value < 0.05 and |log_2_ FC| ≥ log_2_ (1.5) were considered significantly differentially expressed. Detailed sample preparation, LC‐MS/MS acquisition parameters, data processing, and quality‐assessment procedures are provided in the Supporting Methods.

### 4.19 Co‐Immunoprecipitation (Co‐IP) Assays

HEK293 cells were co‐transfected with plasmids encoding FLOT1‐Myc (or empty vector‐Myc) and GST‐α‐Syn using polyethylenimine. At 48 h post‐transfection, cells were harvested and lysed with IP lysis buffer (Beyotime Biotechnology, P0013J) containing a protease inhibitor cocktail. The supernatants were incubated with anti‐c‐Myc magnetic beads (MedChemExpress, HY‐K0206) overnight at 4°C. The beads were washed three times with PBS containing 0.1% Tween‐20, resuspended in 2 × SDS loading buffer, and heated at 100°C for 10 min. After magnetic separation, the eluates were subjected to SDS‐PAGE and immunoblotting with anti‐GST and anti‐Myc antibodies. An anti‐light chain secondary antibody was used to minimize interference from denatured immunoglobulin heavy chains.

### 4.20 Mouse Genotyping

Genomic DNA was extracted from mouse tail using a standard proteinase K digestion and isopropanol precipitation. PCR was performed with gene‐specific primers (forward: 5′‐TCAGCCACGATAAAACTGAGG‐3′; wild type reverse: 5′‐GTGAGGGCTGTGGGTATCTG‐3′; mutant reverse: 5′‐GCCTGAAGAACGAGATCAGC‐3′). PCR products were separated on a 1.5% agarose gel and visualized to determine the genotypes (*Snca* ^+/+^: 105 bp; *Snca* ^*−*/−^: 192 bp).

### 4.21 Statistics

Data are presented as the mean ± SEM. GraphPad Prism (version 10.5.0) was utilized to perform statistical analyses, with each experiment independently repeated at least three times. The Shapiro–Wilk test was employed to assess the normality of the data distribution. For comparisons between two independent groups, an unpaired *t*‐test was applied when the data conformed to a normal distribution; if the variances were unequal, Welch's *t*‐test was used instead. For non‐normally distributed data, the Mann–Whitney *U* test was used as a non‐parametric alternative. For 1‐variable comparisons across multiple groups, a 1‐way ANOVA with Dunnett's post hoc test was applied when variances were homogeneous; in cases of unequal variances, Welch's ANOVA was employed. Multivariate comparisons were conducted with a 2‐way ANOVA followed by Šidák's or Dunnett's multiple comparison test. The significance level (*α*) was set at 0.05. A *p*‐value of less than 0.05 was considered to indicate a statistically significant difference.

## Author Contributions

M.Q. and X.X. performed most experiments, collected all the data, participated in results analysis, visualized data in publication‐ready figures, and wrote the manuscript. X.G., J.C., Y.F., and D.J. contributed to animal experiments and performed clinical atherosclerotic plaque collection. S.W. and J.Y. participated in project discussions and data analysis. L.M. conceived the article, revised the manuscript, and approved the final manuscript. All authors have read and approved the article. M.Q. and X.X. are co‐first authors. The order of co‐first authors was determined by their relative contributions.

## Funding

This work was supported by the National Natural Science Foundation of China (no. 82471355 to Ling Mao).

## Conflicts of Interest

The authors declare no conflicts of interest.

## Supporting information


**Figure S1:** Detection of α‐Syn in human carotid plaques. (a) Representative MS/MS spectra of α‐Syn‐derived peptides identified in human carotid atherosclerotic plaque specimens. (b) Western blot analysis of α‐Syn in human atherosclerotic plaques. The lanes from left to right represent atherosclerotic plaque samples from Patient 1, Patient 2, and Patient 3, followed by recombinant His‐tagged human α‐Syn monomer as a positive control.
**Figure S2:** Genotyping of *Snca* knockout mice. Representative genotyping results are shown for *Snca* ^−/−^ mice and sex‐matched *Snca* ^+/+^ littermate controls. F, female; M, male.
**Figure S3:** *Snca* deficiency similarly reduces atherosclerosis in female mice. (a–c) Representative en face ORO staining of female aortas and aortic arches (a) with quantification of lesion area in the whole aorta (b) and aortic arch (c) (*n* = 6) in female *Snca* ^−/−^ and *Snca* ^+/+^ mice. Scale bar: 5 mm. (d–g) Representative aortic root sections from female mice stained by H&E and ORO (d), with quantification of plaque area (e), necrotic core fraction (f), and lipid deposition at the aortic root lesions (g) (*n* = 6). Scale bar: 200 μm. All data are shown as the mean ± SEM. Statistical analysis was performed using unpaired *t*‐test (b, c, f, g) and Welch's *t*‐test (e). *p < 0.05; ***p < 0.001; ****p < 0.0001.
**Figure S4:** VSMC‐associated lipid accumulation is not detectably altered by *Snca* deletion. (a, b) Representative IF staining of aortic root plaques showing BODIPY and ACTA2 (a), with quantification of VSMC‐derived foam cells (ACTA2^+^ BODIPY^+^) density per mm^2^ plaque area (b) (*n* = 7). Scale bars: 50 μm. All data are shown as the mean ± SEM. Statistical analysis was performed using unpaired *t*‐test. ns, *p* > 0.05.
**Figure S5:** Recombinant His‐α‐Syn with Coomassie brilliant blue staining. Coomassie brilliant blue staining verifying purification of recombinant mouse His‐α‐Syn monomers.
**Figure S6:** α‐Syn affects the mRNA expression of *Lc3b* and *Sqstm1*. RT‐qPCR analysis of *Lc3b* and *Sqstm1* transcripts in RAW264.7 treated with ox‐LDL alone or α‐Syn plus ox‐LDL treatment (*n* = 6). All data are shown as the mean ± SEM. Statistical analysis was performed using unpaired *t*‐test. ns, *p* > 0.05; *p < 0.05.
**Figure S7:** In vivo α‐Syn administration alters autophagy in plaque macrophages. (a, b) Representative plaque IF images and quantification of LC3^+^ CD68^+^ cells per mm^2^ within aortic root lesions from *Apoe* ^−/−^ mice treated with α‐Syn versus PBS (*n* = 7). Scale bar: 100 μm. (c, d) Representative images and quantification of SQSTM1^+^ CD68^+^ cells per mm^2^ in aortic root plaques from *Apoe* ^−/−^ mice treated with α‐Syn versus PBS (*n* = 6). Scale bar: 100 μm. All data are shown as the mean ± SEM. Statistical analysis was performed using unpaired *t*‐test (b, d). *p < 0.05; **p < 0.01.
**Figure S8:** LC‐MS/MS‐based proteomic analysis identifies FLOT1 upregulation in α‐Syn‐treated macrophages. Proteomic analysis identifying FLOT1 as a significantly upregulated protein in α‐Syn‐treated macrophages. Differential protein abundance was assessed using Student's *t*‐tests followed by Benjamini‐Hochberg correction. Proteins with an adjusted *p* value < 0.05 and |log_2_ FC| ≥ log2 (1.5) were considered significantly differentially expressed.
**Figure S9:** Validation of FLOT1 knockdown in RAW264.7 macrophages. Immunoblot analysis confirming the efficiency of FLOT1 knockdown in RAW264.7 cells transfected with *Flot1*‐specific siRNA (si*Flot1*) or non‐targeting control siRNA (si*ctrl*).
**Figure S10:** Colocalization and association between FLOT1 and α‐Syn. (a) Representative confocal IF images of RAW264.7 macrophages treated with 200 nM exogenous α‐Syn for 1 h, followed by staining for FLOT1 and α‐Syn. Scale bar: 10 μm. (b) HEK293 cells were co‐transfected with GST‐tagged α‐Syn and either FLOT1‐Myc or Myc‐tagged empty vector. Cell lysates were immunoprecipitated with an anti‐Myc antibody and analyzed by western blotting using anti‐Myc and anti‐GST antibodies.
**Supporting Methods:** acel70713‐sup‐0001‐SupportingFile.docx.

## Data Availability

The data that support the findings of this study are available from the corresponding author upon reasonable request.
