# Supplementary material for: α‐Synuclein Promotes Atherosclerosis by Impairing Macrophage Autophagic Flux
Source: Aging Cell. 2026 Sep 28;25(10):e70713. doi: 10.1111/acel.70713 (PMC13620287; doi:10.1111/acel.70713)
Supplement: Supplementary file 1 [file ACEL-25-e70713-s001.docx]

**α-Synuclein Promotes Atherosclerosis by Impairing Macrophage Autophagic Flux**

Mengting Qin^#1^, Xuying Xiang^#2^, Xiaoqing Guo^1^, Shengnan Wang^1^, Jiaojiao Chen^1^, Dailiang Jiang^1^, Yuhang Feng^1^, Jiangnan Yu^1^, Ling Mao^1*^

^1^Department of Neurology, Union Hospital, Tongji Medical College, Huazhong University of Science and Technology, Wuhan, China

^2^Department of Rehabilitation, Union Hospital, Tongji Medical College, Huazhong University of Science and Technology, Wuhan, China.

^#^ Mengting Qin and Xuying Xiang contributed equally.

^*^ Correspondence to: Ling Mao, Department of Neurology, Union Hospital, Tongji Medical College, Huazhong University of Science and Technology, Wuhan, China. Email: [maoling@mail.hust.edu.cn](mailto:maoling@mail.hust.edu.cn)

**Contents:**

Figures S1-S10

Methods: Liquid chromatography-tandem mass spectrometry (LC-MS/MS)

**Figure S1. Detection of α-Syn in human carotid plaques.**

(a) Representative MS/MS spectra of α-Syn-derived peptides identified in human carotid atherosclerotic plaque specimens. (b) Western blot analysis of α-Syn in human atherosclerotic plaques. The lanes from left to right represent atherosclerotic plaque samples from Patient 1, Patient 2, and Patient 3, followed by recombinant His-tagged human α-Syn monomer as a positive control.

**Figure S2 Genotyping of *Snca* knockout mice.**

Representative genotyping results are shown for *Snca*^-/-^ mice and sex-matched *Snca^+^*^/+^ littermate controls. F, female; M, male.

**Figure S3. *Snca* deficiency similarly reduces atherosclerosis in female mice.**

(a-c) Representative en face ORO staining of female aortas and aortic arches (a) with quantification of lesion area in the whole aorta (b) and aortic arch (c) (n=6) in female *Snca*^−/−^ and *Snca*^+/+^ mice. Scale bar: 5 mm. (d-g) Representative aortic root sections from female mice stained by H&E and ORO (d), with quantification of plaque area (e), necrotic core fraction (f), and lipid deposition at the aortic root lesions (g) (n=6). Scale bar: 200 μm. All data are shown as the mean ± SEM. Statistical analysis was performed using unpaired t-test (b, c, f, g) and Welch’s t-test (e). *P < 0.05; ***P < 0.001; ****P < 0.0001.

**Figure S4. VSMC-associated lipid accumulation is not detectably altered by *Snca* deletion.**

(a, b) Representative IF staining of aortic root plaques showing BODIPY and ACTA2 (a), with quantification of VSMC-derived foam cells (ACTA2^+^BODIPY^+^) density per mm^2^ plaque area (b) (n=7). Scale bars: 50 μm. All data are shown as the mean ± SEM. Statistical analysis was performed using unpaired t-test. ns, P > 0.05.

**Figure S5. Recombinant His-α-Syn with Coomassie brilliant blue staining.**
Coomassie brilliant blue staining verifying purification of recombinant mouse His-α-Syn monomers.

**Figure S6. α-Syn affects the mRNA expression of *Lc3b* and *Sqstm1***

RT-qPCR analysis of *Lc3b* and *Sqstm1* transcripts in RAW264.7 treated with ox-LDL alone or α-Syn plus ox-LDL treatment (n=6). All data are shown as the mean ± SEM. Statistical analysis was performed using unpaired t-test. ns, P > 0.05; *P < 0.05.

**Figure S7. In vivo α-Syn administration alters autophagy in plaque macrophages.**

(a, b) Representative plaque IF images and quantification of LC3^+^CD68^+^ cells per mm² within aortic root lesions from *Apoe*^−/−^ mice treated with α-Syn versus PBS (n=7). Scale bar: 100 μm. (c, d) Representative images and quantification of SQSTM1^+^CD68^+^ cells per mm² in aortic root plaques from *Apoe*^−/−^ mice treated with α-Syn versus PBS (n=6). Scale bar: 100 μm. All data are shown as the mean ± SEM. Statistical analysis was performed using unpaired t-test (b, d). *P < 0.05; **P < 0.01.

**Figure S8. LC-MS/MS-based proteomic analysis identifies FLOT1 upregulation in α-Syn-treated macrophages.**
Proteomic analysis identifying FLOT1 as a significantly upregulated protein in α-Syn-treated macrophages. Differential protein abundance was assessed using Student’s t-tests followed by Benjamini-Hochberg correction. Proteins with an adjusted P value < 0.05 and |log2 FC| ≥ log2 (1.5) were considered significantly differentially expressed.

**Figure S9. Validation of FLOT1 knockdown in RAW264.7 macrophages.**

Immunoblot analysis confirming the efficiency of FLOT1 knockdown in RAW264.7 cells transfected with *Flot1*-specific siRNA (si*Flot1*) or non-targeting control siRNA (si*ctrl*).

**Figure S10. Colocalization and association between FLOT1 and α-Syn.**

(a) Representative confocal IF images of RAW264.7 macrophages treated with 200 nM exogenous α-Syn for 1 h, followed by staining for FLOT1 and α-Syn. Scale bar: 10 μm. (b) HEK293 cells were co-transfected with GST-tagged α-Syn and either FLOT1-Myc or Myc-tagged empty vector. Cell lysates were immunoprecipitated with an anti-Myc antibody and analysed by western blotting using anti-Myc and anti-GST antibodies.

**Liquid chromatography-tandem mass spectrometry (LC-MS/MS)**

**Proteomic analysis of human carotid plaques**

Human carotid plaque tissues were lysed in RIPA lysis buffer (Beyotime, China) supplemented with a protease inhibitor cocktail. Protein samples (100 µg) were sequentially reduced with 10 mM TCEP (37°C, 60 min) and alkylated with 40 mM IAM (room temperature, dark, 40 min). Proteins were precipitated with pre-chilled acetone (6:1, v/v) at -20°C for 4 h, followed by centrifugation at 10,000 × g for 20 min. The pellets were dissolved in 100 µL of 100 mM TEAB and digested overnight at 37°C with trypsin (1:50, w/w). The resulting peptides were desalted using HLB and MCX cartridges and quantified by UV spectrophotometry using a NanoDrop One spectrophotometer (Thermo Fisher Scientific, USA) prior to LC-MS/MS analysis. LC-MS/MS analysis was performed on a timsTOF HT mass spectrometer (Bruker, Germany) coupled with a Vanquish Neo liquid chromatography system (Thermo, USA). Peptides were separated on an in-house packed analytic column (15 cm x 100 µm, 1.7 µm) at a flow rate of 300 nL/min over an 8-min gradient. Mobile phase A consisted of 98% water, 2% acetonitrile, and 0.1% formic acid, while mobile phase B consisted of 80% acetonitrile, 20% water, and 0.1% formic acid. Peptides were separated using an 8-min linear gradient from 4% to 99% mobile phase B at a flow rate of 300 nL/min.

LC-MS/MS data were acquired in data-independent acquisition mode using Compass HyStar software (Bruker, Germany). The electrospray voltage was set to 1.5 kV. Full MS1 scans were acquired over an m/z range of 300-1500, and fragment-ion spectra were recorded over an m/z range of 100-1700, with a nominal resolving power of approximately 60,000. DIA data were collected using a dia-PASEF acquisition scheme. The ion mobility scan range was set to 0.60-1.60 V·s·cm⁻² (1/K₀), with an ion mobility ramp time of 50 ms and a target intensity of 5,000. The collision energy was varied from 20 to 59 eV as a function of ion mobility. The dia-PASEF acquisition scheme consisted of 12 ramps with variable precursor isolation windows, collectively covering approximately m/z 357-1205; the mobility-dependent isolation windows covered approximately 0.69-1.30 V·s·cm⁻² (1/K₀). No dynamic exclusion was applied. Raw DIA data were analysed using the library-free directDIA workflow implemented in Spectronaut (version 19) against the UniProtKB Homo sapiens reference proteome database (taxonomy ID: 9606). Peptide and protein identifications were filtered at a false discovery rate (FDR) of 1% at both the peptide and protein levels. For the quantification of specific peptides, extracted ion chromatograms (XICs) of the corresponding precursor ions were generated using an XIC extraction width of ≤ 75 ppm.

For α-synuclein-specific peptides, precursor-level, chromatographic, fragment-ion, and mass-accuracy information was exported from Spectronaut and manually inspected. Peptide sequence, precursor charge and calibrated m/z, peak start and apex retention times, precursor-level quantitative values, fragment-ion annotations and peak areas, and fragment-ion mass errors were evaluated to support peptide identification. The observed mass errors were compared with the corresponding software-defined tolerances.

**Proteomic analysis of RAW264.7 cells**

After the indicated treatments, RAW264.7 cells were harvested and lysed in RIPA lysis buffer. Proteins were extracted, quantified by BCA, and equal amounts were reduced, alkylated, and digested with trypsin overnight. Peptides were desalted, vacuum-dried, and analyzed by LC-MS/MS on an UltiMate 3000 RSLCnano system (Thermo, USA) coupled to a timsTOF Pro mass spectrometer (Bruker, Germany). Peptides were trapped on a C18 trap column (75 µm x 2 cm, 3 µm) and separated on a reversed-phase C18 analytical column (75 µm x 15 cm, 1.7 µm) at 300 nL/min using a gradient of mobile phase A (0.1% formic acid in water) and B (0.1% formic acid in acetonitrile). The mass spectrometer was operated in dia-PASEF mode with capillary voltage of 1500 V. The MS and MS/MS spectra were acquired from 100 to 1700 m/z. The ion mobility range was set to 0.6-1.6 V·s·cm⁻² (1/K₀), with an ion mobility ramp time of 50 ms. Collision energy was ramped from 20 to 59 eV as a function of ion mobility. Data were processed with DIA-NN (v1.8.1) software in library-free mode against the Mus musculus UniProt database. Precursor- and protein-level identifications were controlled at FDR of 1% at both levels. Bioinformatic analyses were conducted in R. Missing values were imputed by random sampling from a left-shifted Gaussian distribution, with a downshift of 1.8 standard deviations and a width of 0.25 standard deviations, to simulate signals near the detection limit. Proteins with ≥50% valid values in at least one group were retained. Differentially expressed proteins (DEPs) were identified based on log_2_ fold change (FC) and Student's t-test. The P values were adjusted using the Benjamini-Hochberg method to control the FDR. Proteins with an adjusted P value < 0.05 and a |log_2_ FC| ≥ log_2_(1.5) (approximately 0.585) were considered significantly differentially expressed and visualized by volcano plots. LC-MS/MS analysis and proteomic data processing were performed with technical support from SpecAlly Life Technology Co., Ltd (Wuhan, China).
